# Supplementary material for: DDX6 Helicase Behavior and Protein Partners in Human Adipose Tissue-Derived Stem Cells during Early Adipogenesis and Osteogenesis
Source: Int J Mol Sci. 2020 Apr 9;21(7):2607. doi: 10.3390/ijms21072607 (PMC7177724; doi:10.3390/ijms21072607)
Supplement: Supplementary file 1 [file ijms-21-02607-s001.zip › Supplementary Figures.pdf]

Supplementary Figures

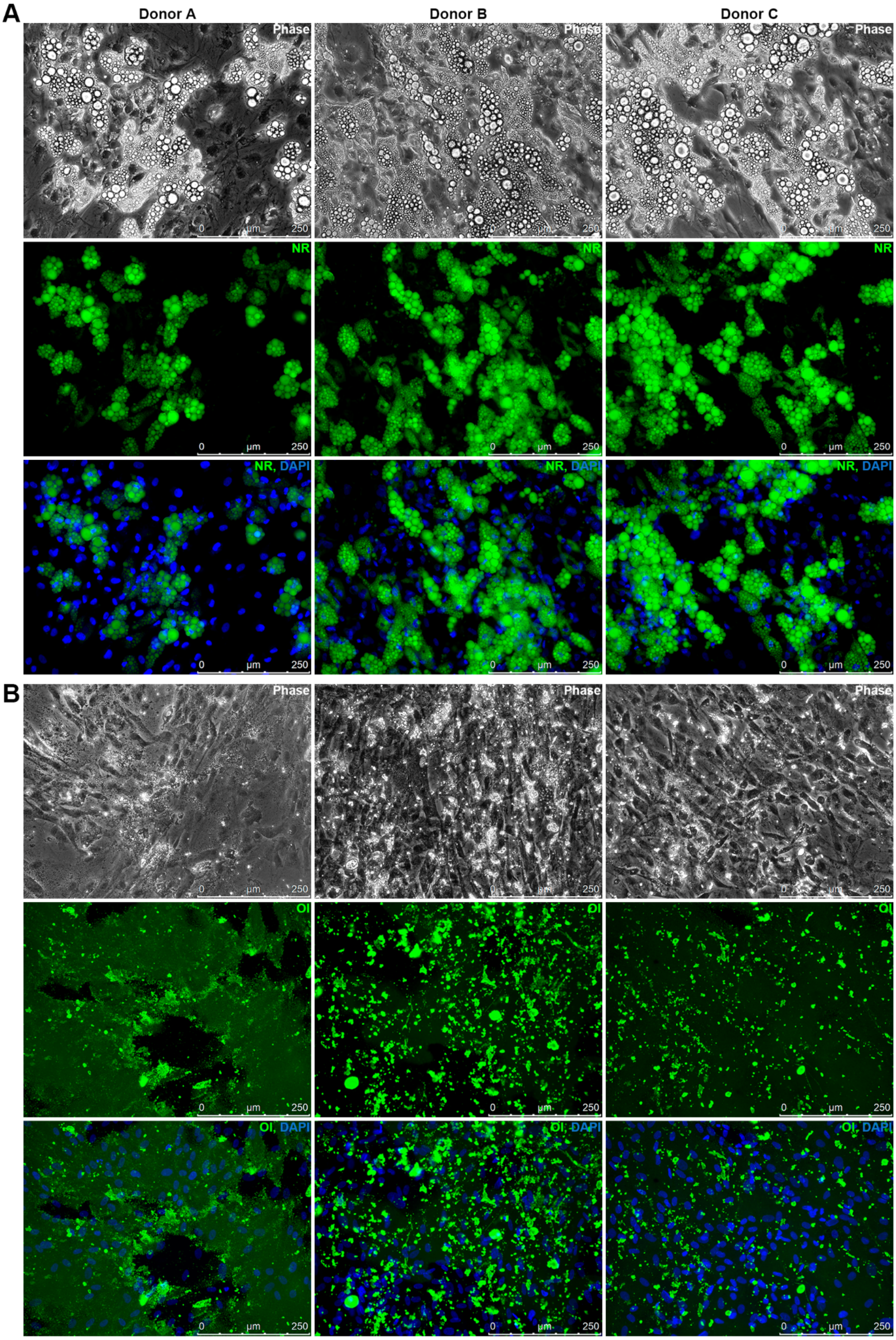

Figure S1. Results from the analysis of the differentiation potential of the hASCs. (**A**) Microscopy analysis of the hASCs induced to adipogenesis for 28 days. After treatment, lipid droplets, which are characteristic of adipocytes, can be visualized by phase contrast and are stained green by Nile red.

Nuclei were stained with DAPI. (**B**) Results from the microscopy analysis of the hASCs induced to osteogenesis for 21 days. After treatment, OsteoImage Mineralization Assay was used to detect bone-like nodules in the cell cultures. The kit consists of a fluorescent green staining reagent that specifically binds to the hydroxyapatite portion of these nodules. Nuclei were stained with DAPI.

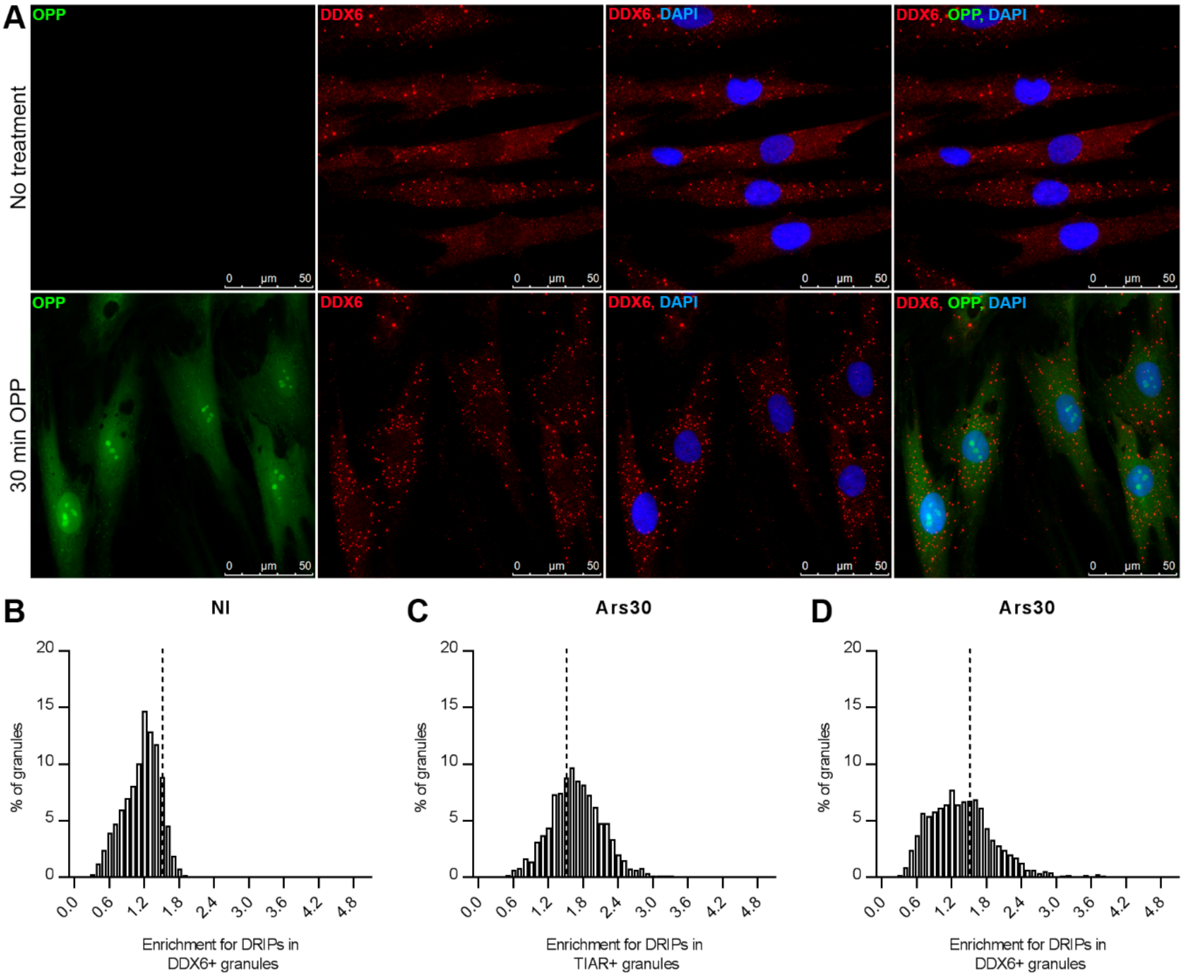

Figure S2. DDX6 granule assembly was stimulated by OPP treatment and only accumulated DRIPs after stress induction. (**A**) Results from immunofluorescence analysis with microscopy of DDX6 and DRIP localization in the hASCs not treated or treated with OPP. Nuclei were stained with DAPI. (**B**) Frequency distribution histogram (percentage) of DRIP enrichment in the DDX6 granules found in the hASCs treated only with OPP for 30 min. (**C**) Frequency distribution histogram (percentage) of DRIP enrichment in the TIAR granules found in the hASCs treated with OPP and sodium arsenite for 30 min. (**D**) Frequency distribution histogram (percentage) of DRIP enrichment in the DDX6 granules found in the hASCs treated with OPP and sodium arsenite for 30 min.

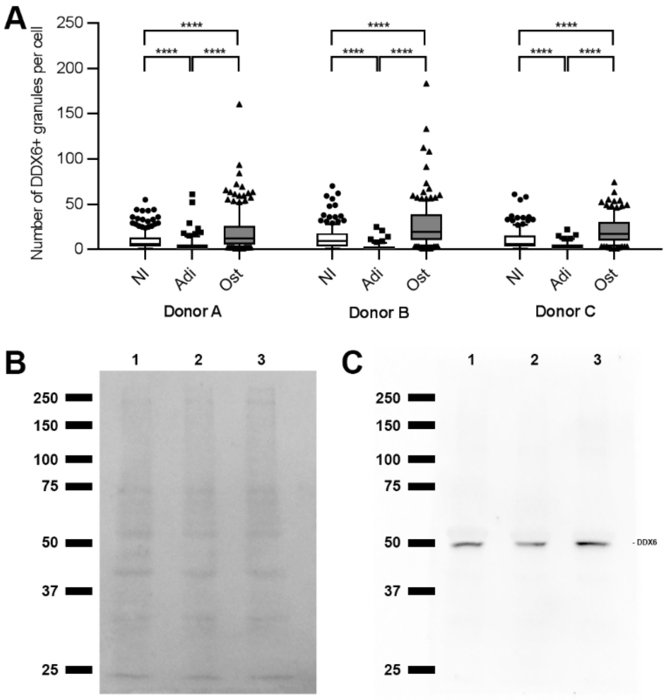

Figure S3. Quantification of the DDX6 protein and granules in the hASCs not inducted or induced to adipogenesis or osteogenesis for 24 h. (**A**) Number of DDX6 granules per cell in the hASCs maintained in noninductive medium (NI) or induced to adipogenesis (Adi) or osteogenesis (Ost) for 24 h. The graph shows the results obtained for each donor; quartile 10-90%; Kruskal-Wallis test with Dunn's multiple comparisons posttest: * $p < 0.05$. (**B**) Western blot analysis results of DDX6 expression in the hASCs maintained in noninductive medium (1) or induced to adipogenesis (2) or osteogenesis (3) for 24 h. Representative image of the membrane with the proteins stained with Ponceau. Molecular weight is presented in kDa. (**C**) Western blot analysis results of DDX6 expression in the hASCs maintained in noninductive medium (1) or induced to adipogenesis (2) or osteogenesis (3) for 24 h. Representative image of the membrane submitted to labeling with anti-DDX6 antibody. Molecular weight is presented in kDa.

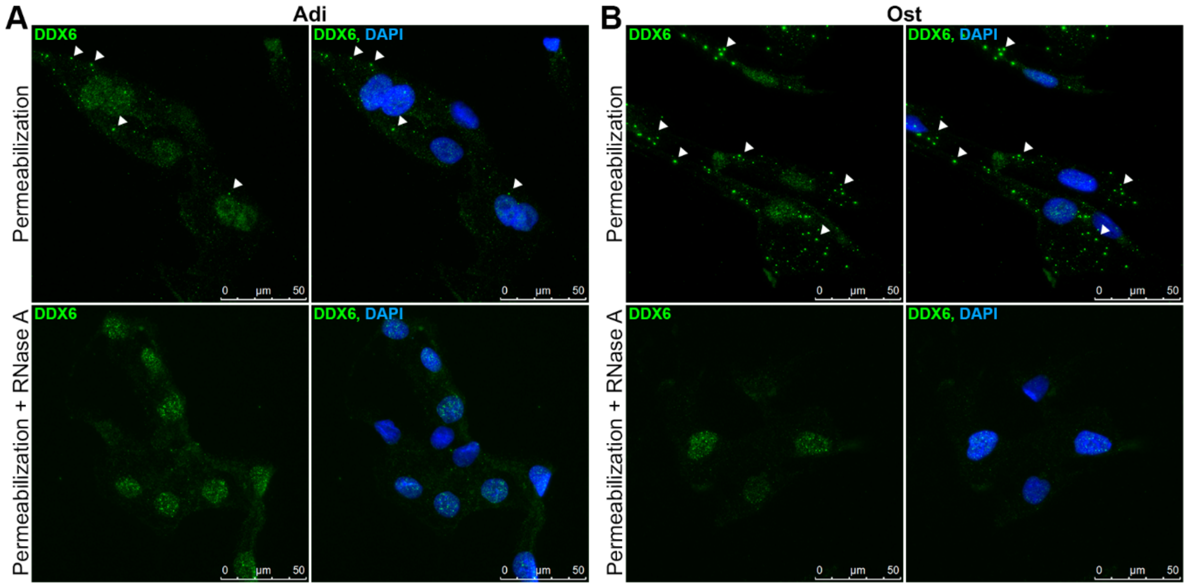

Figure S4. hASCs induced to adipogenesis or osteogenesis for 24 h showing RNA-dependent DDX6 granules. (**A**) Results from the immunofluorescence analysis of DDX6 in the hASCs treated with adipogenic induction medium for 24 h and then permeabilized or permeabilized and treated with RNase A. After RNase treatment, the DDX6 granules were disassembled. (**B**) Results from the immunofluorescence analysis of DDX6 in the hASCs treated with osteogenic induction medium for

24 h and then permeabilized or permeabilized and treated with RNase A. After RNase treatment, the DDX6 granules were disassembled. White arrows: granules containing DDX6. Nuclei were stained with DAPI.

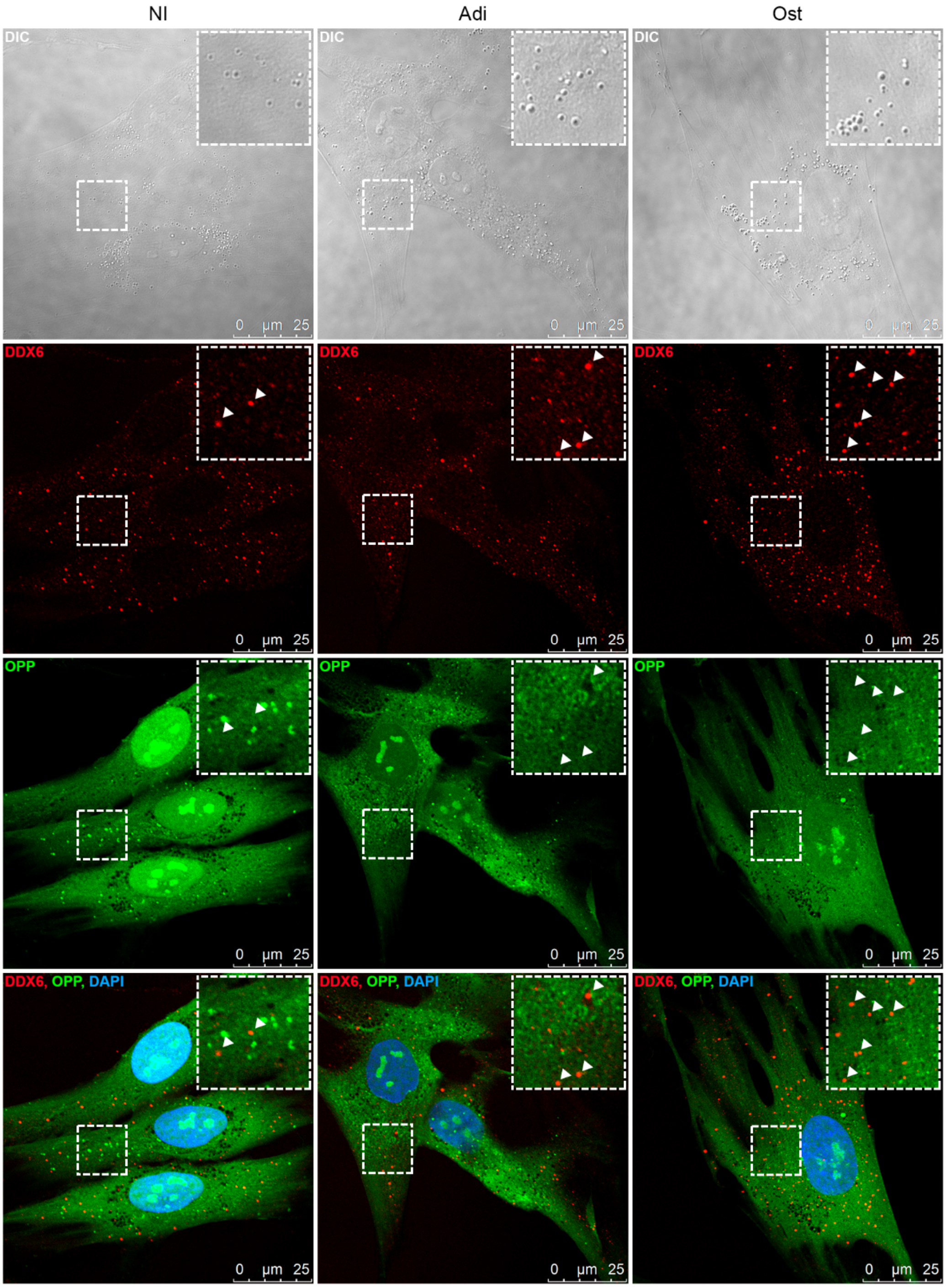

Figure S5. DDX6 granules in the hASCs do not accumulate DRIPs after adipogenic or osteogenic differentiation induction. Results from immunofluorescence analysis with confocal microscopy of DDX6 and DRIP localization in the hASCs maintained in noninductive medium or induced to adipogenesis or osteogenesis for 24 h. White arrows: DDX6 granules. Nuclei were stained with DAPI.

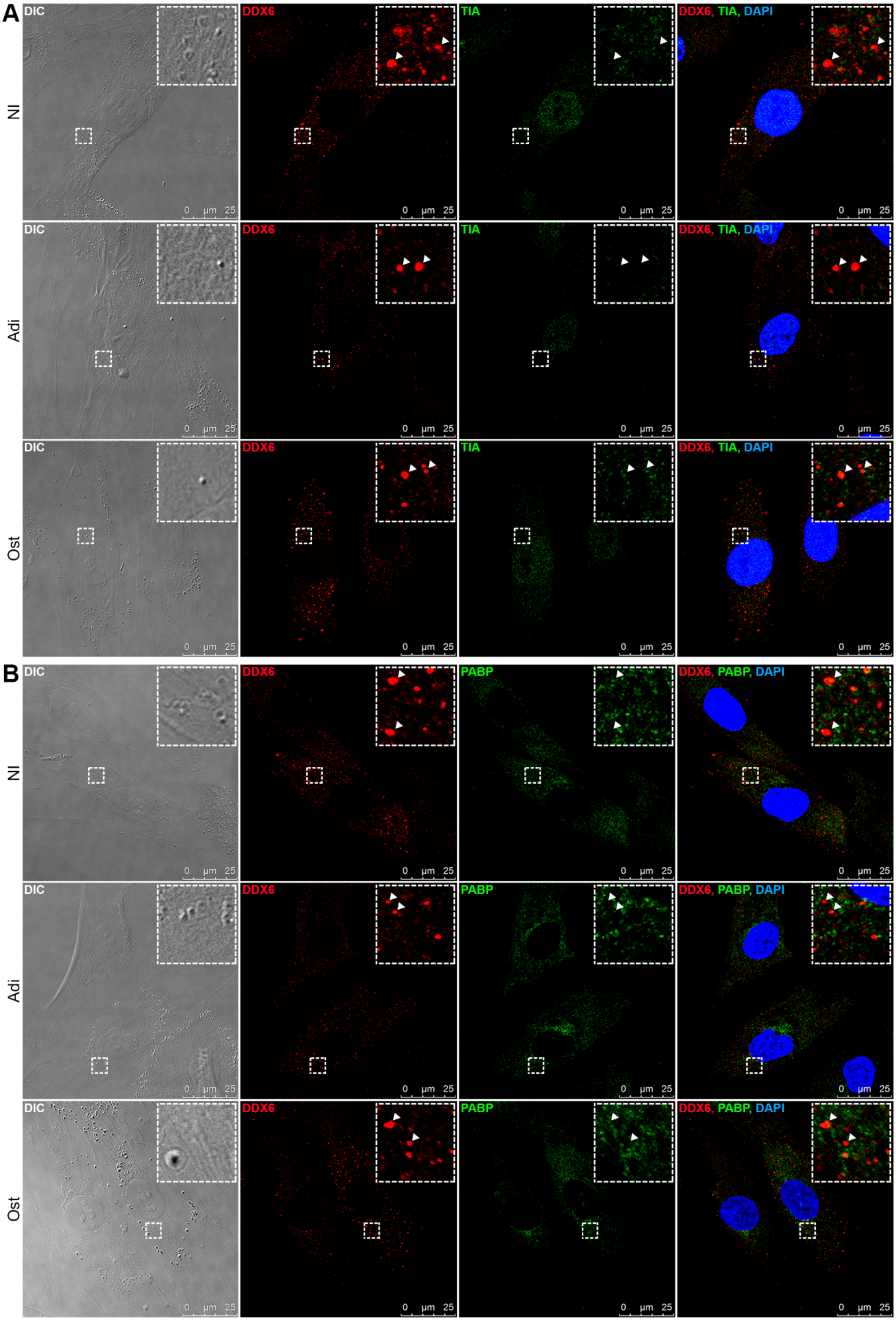

A
B
DIC
DDX6
TIA
DDX6, TIA, DAPI
PABP
DDX6, PABP, DAPI
NI
Adi
Ost
0 μm 25

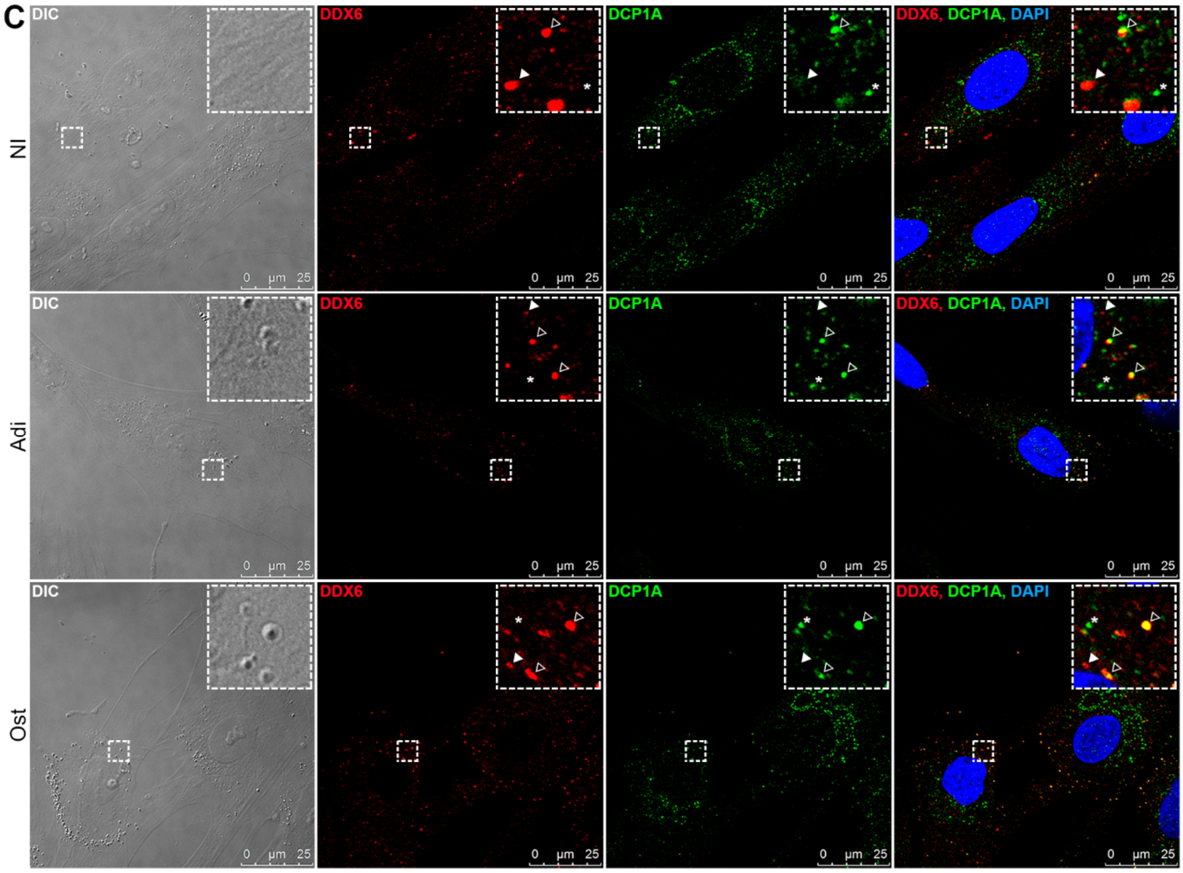

Figure S6. Localization of DDX6 with SG and P-body characteristic proteins in the hASCs after adipogenic or osteogenic induction. The distribution of the proteins in the hASCs that were not induced (NI) or induced to adipogenesis (Adi) or osteogenesis (Ost) for 24 h was analyzed by immunofluorescence with confocal microscopy. (**A**) TIA1 was found in the nucleus of the hASCs under NI, Adi and Ost conditions and did not colocalize with the DDX6 granules. (**B**) PABP was found in the cytoplasm of the hASCs under NI, Adi and Ost conditions but did not accumulate with the DDX6 granules. (**C**) In the hASCs under NI, Adi and Ost conditions, DDX6 partially colocalized with DCP1A. White arrows: granules that contain only DDX6; asterisks: granules that contain only DCP1A (C), Hollow arrows: granules containing both DDX6 and DCP1A (C). Nuclei were stained with DAPI.

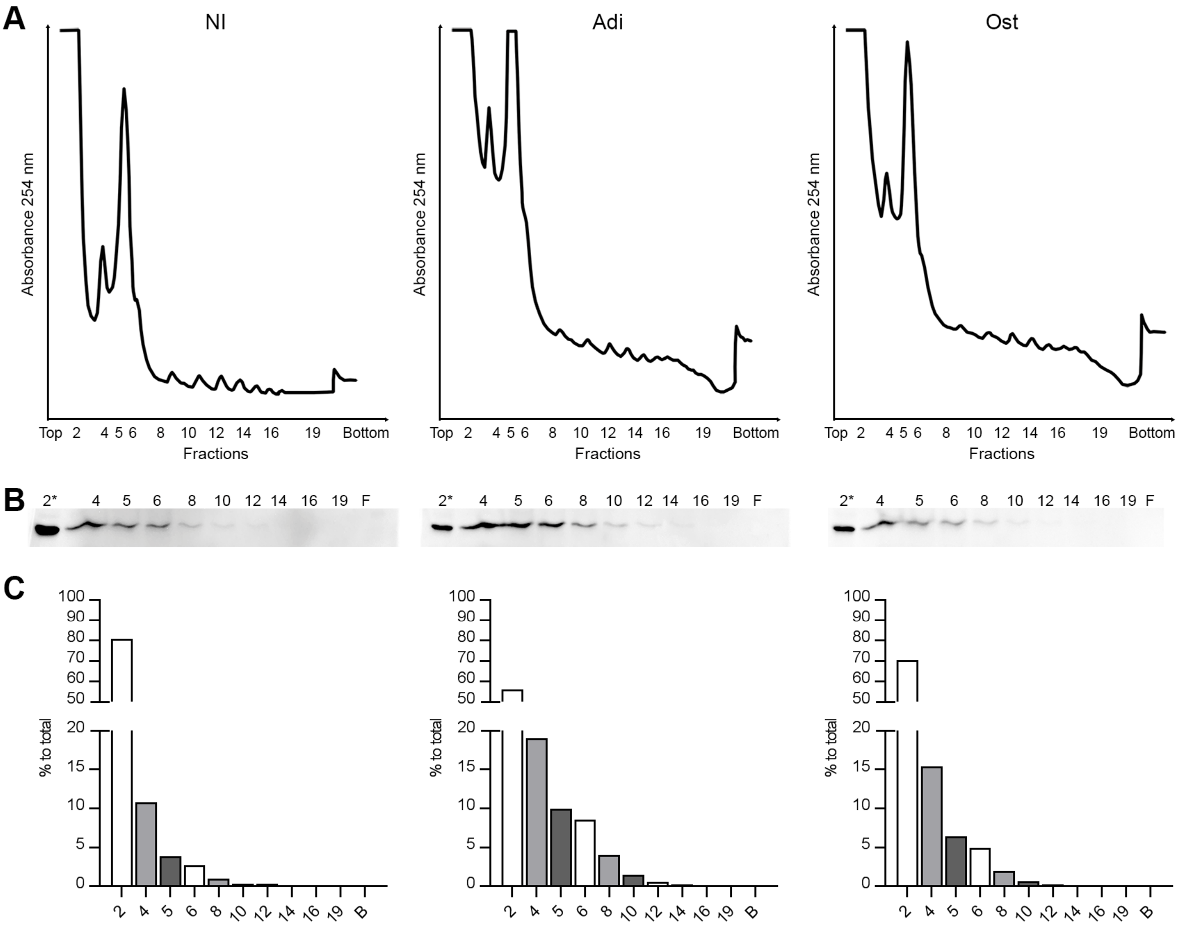

Figure S7. DDX6 distribution in the sucrose gradient. (**A**) Polysomal profile from the cell extracts of the hASCs not induced or induced to adipogenesis or osteogenesis for 24 h. (**B**) Results from the Western blot analysis for DDX6 and different fractions of the sucrose density gradient. Fraction 2: ribosome-free; fractions 4, 5, and 6: monosomes; fractions 8, 10 and 12: light polysomes; fractions 14, 16 and 19: heavy polysomes; and bottom of the gradient. * For Western blot analysis, fraction 2 was diluted 1:5. (**C**) Quantification of the DDX6 found in the different fractions. The bar graph represents the ratio of the signal obtained for each fraction to the total signal.

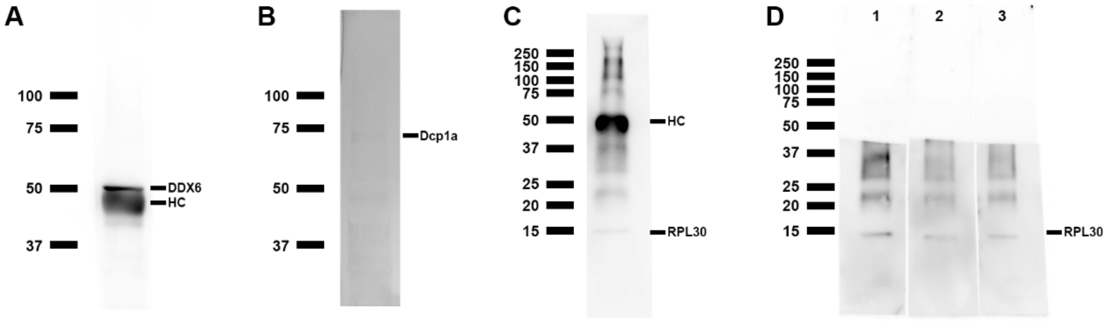

Figure S8. Western blot analysis of the proteins found in DDX6 immunoprecipitation. Western blot analysis using the protein extract from DDX6 immunoprecipitation from hASCs kept in noninductive medium and submitted to labeling with (**A**) anti-DDX6, (**B**) anti-Dcp1a and (**C**) anti-RPL30 antibodies. (**D**) Western blot analysis using the protein extract from DDX6 immunoprecipitation from hASCs maintained in noninductive medium (1) or induced to adipogenesis (2) or osteogenesis (3) for 24 h and submitted to labeling with anti-DDX6. Molecular weight is presented in kDa.

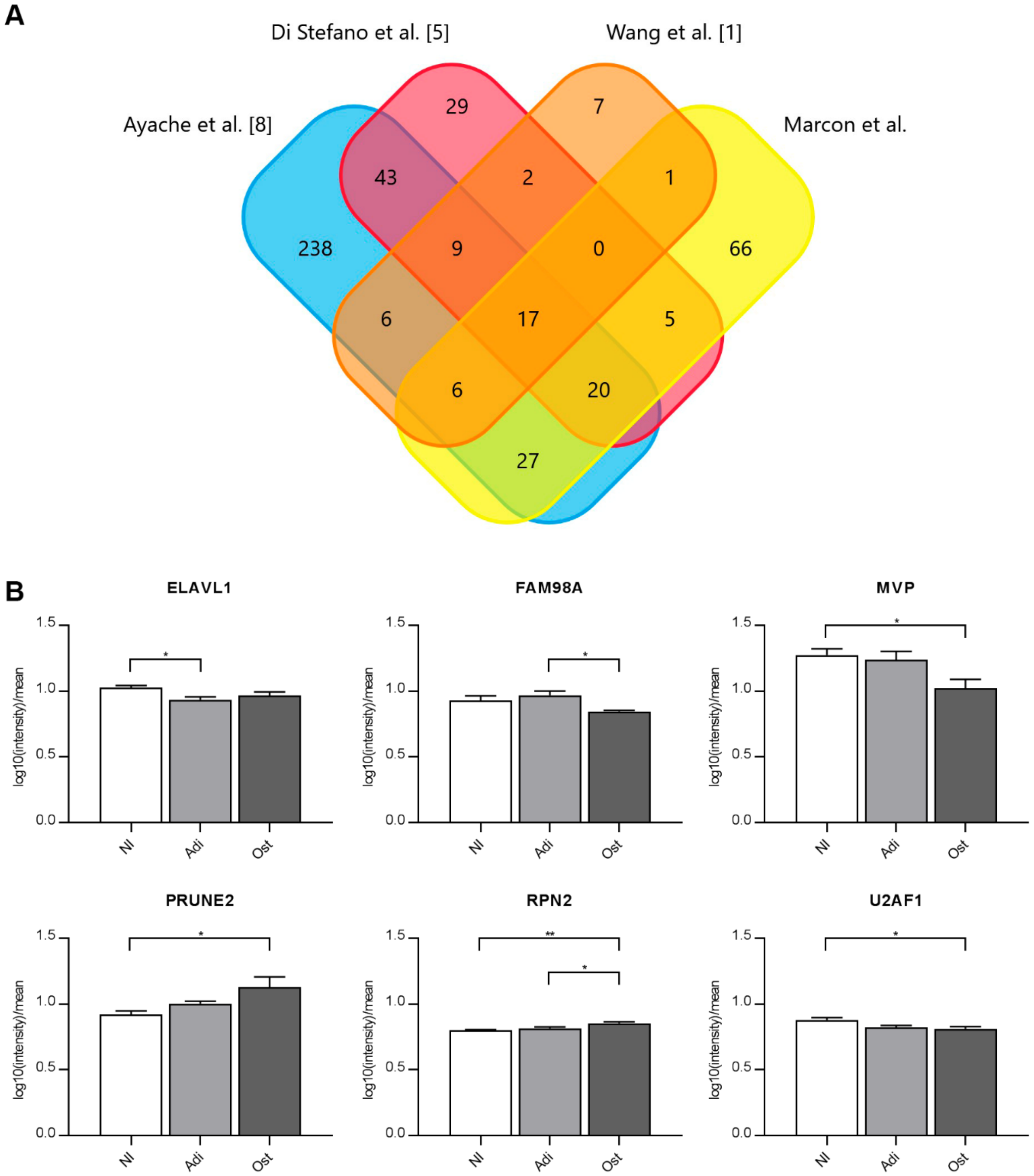

Figure S9. Results of the analysis of the proteins identified by immunoprecipitation with DDX6 from the hASCs: comparison with other cell types and identification of the proteins differentially enriched under each condition. (**A**) Qualitative comparison of proteins identified by immunoprecipitation in the hASCs and in other cell types. The comparisons were performed with HEK293T cells (from the study of Ayache and collaborators [8]), hiPSCs (from the study of DiStefano and collaborators [5]) and epidermal progenitors (from the work of Wang and collaborators [1]). (**B**) Proteins with different levels of enrichment in the DDX6 coimmunoprecipitation fraction from the hASCs not induced (NI) or induced to adipogenesis (Adi) or osteogenesis (Ost) for 24 h. Mean with SEM. One-way ANOVA with multiple comparisons and Tukey's post hoc test: * $p < 0.05$ and ** $p < 0.01$.
