# Supplementary material for: DDX6 Helicase Behavior and Protein Partners in Human Adipose Tissue-Derived Stem Cells during Early Adipogenesis and Osteogenesis
Source: Int J Mol Sci. 2020 Apr 9;21(7):2607. doi: 10.3390/ijms21072607 (PMC7177724; doi:10.3390/ijms21072607)
Supplement: Supplementary file 1 [file ijms-21-02607-s001.zip › Supplementary Table S1.pdf]

Table S1. Immunophenotypic profile of the hASCs used in this study.

Phenotype	Marker of	Donor A (P4)	Donor B (P5)	Donor C (P5)
CD90+	mesenchymal stem cells	98,42%	92,02%	98,45%
CD105+	mesenchymal stem cells	99,34%	99,80%	99,92%
CD73+	mesenchymal stem cells	99,39%	99,90%	99,89%
CD140b+	mesenchymal stem cells derived	99,90%	93,70%	99,91%
CD34+	hematopoietic progenitor	5,68%	2,39%	3,03%
CD11b+	macrophages and monocytes	1,20%	2,19%	1,48%
CD45+	hematopoietic cells	0,73%	1,98%	1,39%
CD19+	B cells	1,49%	1,95%	2,27%
HLADR+	leucocytes	3,91%	3,13%	4,56%
CD31+	endothelial cells	1,31%	2,02%	1,50%
